# Magnetoelectric Effect in Ceramics Based on Bismuth Ferrite

**DOI:** 10.1186/s11671-016-1436-3

**Published:** 2016-04-30

**Authors:** Elżbieta Jartych, Tomasz Pikula, Karol Kowal, Jolanta Dzik, Piotr Guzdek, Dionizy Czekaj

**Affiliations:** Institute of Electronics and Information Technology, Lublin University of Technology, Nadbystrzycka 38a Str., 20-618 Lublin, Poland; Institute of Technology and Mechatronics, University of Silesia, Żytnia 12 Str., 41-200 Sosnowiec, Poland; Institute of Electron Technology, Cracow Division, Zabłocie 39 Str., 30-701 Kraków, Poland

**Keywords:** Multiferroics, Ceramic materials, Bismuth ferrite, Aurivillius compounds, Magnetoelectric effect

## Abstract

Solid-state sintering method was used to prepare ceramic materials based on bismuth ferrite, i.e., (BiFeO_3_)_1 − *x*_–(BaTiO_3_)_*x*_ and Bi_1 − *x*_Nd_*x*_FeO_3_ solid solutions and the Aurivillius Bi_5_Ti_3_FeO_15_ compound. The structure of the materials was examined using X-ray diffraction, and the Rietveld method was applied to phase analysis and structure refinement. Magnetoelectric coupling was registered in all the materials using dynamic lock-in technique. The highest value of magnetoelectric coupling coefficient *α*_ME_ was obtained for the Bi_5_Ti_3_FeO_15_ compound (*α*_ME_ ~ 10 mVcm^−1^ Oe^−1^). In the case of (BiFeO_3_)_1 − *x*_–(BaTiO_3_)_*x*_ and Bi_1 − *x*_Nd_*x*_FeO_3_ solid solutions, the maximum *α*_ME_ is of the order of 1 and 2.7 mVcm^−1^ Oe^−1^, respectively. The magnitude of magnetoelectric coupling is accompanied with structural transformation in the studied solid solutions. The relatively high magnetoelectric effect in the Aurivillius Bi_5_Ti_3_FeO_15_ compound is surprising, especially since the material is paramagnetic at room temperature. When the materials were subjected to a preliminary electrical poling, the magnitude of the magnetoelectric coupling increased 2–3 times.

## Background

Multiferroics are the class of intelligent materials which exhibit at least two from the three possible *ferro*- orders, i.e., ferromagnetic (also ferrimagnetic, antiferromagnetic, or ferrotoroidal), ferroelectric, and ferroelastic [1, 2]. Especially interesting from the application point of view is the group of magnetoelectrics which offers long-range ordering of the elementary magnetic moments as well as long-range ordering of the electric dipoles [3, 4]. Moreover, they exhibit a coupling between the magnetic and the electric sub-systems what makes possible induction of magnetization by applying an external electric field or induction of electric polarization by applying an external magnetic field [5, 6]. Therefore, the so-called magnetoelectric (ME) effect attracts a lot of interest also from the basic research point of view. Experimentally, ME effect is measured and expressed by magnetoelectric coupling coefficient, *α*_ME_, typically specified in units of mVcm^−1^ Oe^−1^.

Although the magnetoelectric effect is known since the 1960s of the last century, an intensive growth of interest in the possibilities of practical application of this phenomenon occurred in the late 1990s. In 2003 and 2004, the first results confirming the possibility of switching electrical polarization by an external magnetic field in TbMnO_3_ and TbMn_2_O_5_ were published [7, 8]. Although this effect has been observed only at low temperatures, these works gave rise to many new research projects focusing on the *α*_ME_ optimization. The higher value of this parameter can be achieved in magnetoelectric material at room temperature, and the wider range of its potential applications can be expected. The most frequently mentioned prototypes of magnetoelectric devices which were developed so far are magnetic field sensors [9–11] and energy harvesting devices [12–14]. Among the new proposals for the use of magnetoelectric coupling phenomenon, the most spectacular and forward-looking ideas seem to be new-generation memories [15–17], spintronic devices (e.g., spin valves, magnetic tunnel junctions) [18–20], microwave, millimeter-wave devices and miniature antennas [14], and wireless medical tools (e.g., for endoscopy and brain imaging) [14]. The implementation of such innovative devices requires, however, the elaboration of materials having possibly high magnetoelectric coupling coefficient.

The magnetoelectric effect is especially high in composite materials. In particulate composites, like BaTiO_3_/CoFe_2_O_4_, PbZr_1 − *x*_Ti_*x*_O_3_(PZT)/Tb_1 − *x*_Dy_*x*_Fe_2_(Terfenol-D), and Ba_0.8_Pb_0.2_TiO_3_/CuFe_1.8_Cr_0.2_O_4_, the value of *α*_ME_ is of the order of 100–130 mVcm^−1^ Oe^−1^ [6]. The laminated composites exhibit the largest value of *α*_ME_, e.g., in PZT/Terfenol-D laminates, ME response achieves ~4.7 Vcm^−1^ Oe^−1^ [21] or even 90 Vcm^−1^ Oe^−1^ in laminated PZT/Permendur composites [22]. The magnetoelectric coupling is also observed in nanostructured multiferroic ceramic materials (e.g., YMnO_3_ [23]), semiconducting membranes galvanically filled with a magnetostrictive material (e.g., InP membrane filled with Ni [24]) as well as in nanocomposite thin films (e.g., CoFe_2_O_4_ polymer nanocomposite thin films [25]).

The synthesis of new, single-phase, multiferroic materials which exhibit high value of the magnetoelectric coupling coefficient is still a challenge. Until now, the best recognized single-phase multiferroic compound is bismuth ferrite BiFeO_3_ in which the ferroelectric and antiferromagnetic ordering coexist at ambient temperature (antiferromagnetic Néel temperature *T*_N_ = 643 K, ferroelectric Curie temperature *T*_C_ = 1100 K) [26]. However, due to the cycloidal modulation of spin arrangement, the linear magnetoelectric effect in bismuth ferrite in the form of bulk polycrystalline sample is not observed. In thin films of BiFeO_3_, where spin cycloid disappears, a giant magnetoelectric coupling was observed, *α*_ME_ ~ 3 Vcm^−1^ Oe^−1^, as reported in [27]. Therefore, many current investigations tend to destroy the spin cycloid and to release the inherent magnetization in order to improve multiferroic properties of BiFeO_3_. This may be achieved, e.g., by structural modifications or deformations introduced by cation substitution or doping or creating of solid solutions of BiFeO_3_ with other materials with ABO_3_ type of structure.

In the present work, three types of ceramic materials based on bismuth ferrite BiFeO_3_ were prepared by solid-state sintering method, i.e., (BiFeO_3_)_1 − *x*_–(BaTiO_3_)_*x*_ solid solutions, bismuth ferrite doped by neodymium Bi_1 − *x*_Nd_*x*_FeO_3_, and the Aurivillius Bi_5_Ti_3_FeO_15_ compound. The structure and selected magnetic and electric properties of these materials have been already investigated and reported [28–32]. However, one can note that there is lack of data of direct measurements of magnetoelectric coupling in materials based on the bismuth ferrite. Dynamic lock-in technique was adopted in the present study to measure the magnetoelectric coupling effect. In contrast to static and quasi-static methods, the dynamic technique allows avoiding errors caused by charge accumulation on the grain boundaries in polycrystalline samples [6, 33]. The value of the magnetoelectric coupling coefficient was determined using formula

1 $$ {\alpha}_{\mathrm{ME}}=\frac{\mathrm{d}E}{\mathrm{d}{H}_{\mathrm{DC}}}=\frac{1}{t}\kern0.24em \frac{V_{\mathrm{OUT}}}{H_{\mathrm{AC}}}, $$

where *V*_OUT_ is the output voltage (induced between the sample surfaces due to the magnetoelectric effect) registered by the lock-in amplifier, *t* is the thickness of the sample (in the form of disk), *H*_DC_ is the magnitude of the DC magnetic field produced by an electromagnet, and *H*_AC_ is the amplitude of the small sinusoidal magnetic field superimposed onto the DC magnetic field [6, 33].

The aim of our studies was to compare the magnitude of magnetoelectric coupling in ceramic materials mentioned above and to determine the influence of the structure on the value of magnetoelectric response of a given material.

## Methods

The conventional solid-state sintering method was used to prepare the samples from the oxides and commercial compounds of 99.9 % purity. In the case of (BiFeO_3_)_1 − *x*_–(BaTiO_3_)_*x*_ solid solutions, the synthesis was performed according to the following reaction:

2 $$ {\left({\mathrm{Bi}}_2{\mathrm{O}}_3\right)}_{\frac{1-x}{2}}+{\left({\mathrm{Fe}}_2{\mathrm{O}}_3\right)}_{\frac{1-x}{2}}+{\left({\mathrm{TiO}}_2\right)}_x+{\left({\mathrm{BaCO}}_3\right)}_x\kern0.36em \to {\left({\mathrm{Bi}\mathrm{FeO}}_3\right)}_{1-x}-{\left({\mathrm{BaTiO}}_3\right)}_x+{\left({\mathrm{CO}}_2\right)}_x. $$

As recently reported, (BiFeO_3_)_1 − *x*_–(BaTiO_3_)_*x*_ solid solutions exhibit maximum magnetoelectric coupling within a narrow composition range, i.e., *x* = 0.2–0.3 which is connected with the structural transformation of the samples from rhombohedral to cubic symmetry [34]. As reported in our earlier work [35], the magnetoelectric properties of (BiFeO_3_)_1 − *x*_–(BaTiO_3_)_*x*_ solid solutions are strongly dependent on the sintering temperature *T* and the highest magnetoelectric effect was observed for the sample with *x* = 0.3 sintered at *T* = 1153 K. Therefore, in current studies after the preliminary synthesis at 1123 K for 2 h, the sintering was performed at temperature *T* = 1153 K for 4 h. After that, the ceramics were annealed in air at a temperature of 823 K for 10 h and then cooled with a linear decrease in temperature (100 K h^−1^). Three samples with the barium titanate concentration *x* = 0.25, 0.4, and 0.5 were prepared in the form of disks with a diameter of 10 mm and 1-mm thickness. The concentrations of *x* = 0.25, 0.4, and 0.5 were selected in order to check whether there was a structural transformation in this range and for which barium titanate contents the maximum magnetoelectric coupling might be observed.

The second series of the samples, i.e., Bi_1 − *x*_Nd_*x*_FeO_3_ solid solutions, was prepared by doping bismuth ferrite by neodymium ions according to the reaction

3 $$ \left(1-x\right){\mathrm{Bi}}_2{\mathrm{O}}_3+x{\mathrm{Nd}}_2{\mathrm{O}}_3+{\mathrm{Fe}}_2{\mathrm{O}}_3\to 2{\mathrm{Bi}}_{1-x}{\mathrm{Nd}}_x{\mathrm{Fe}\mathrm{O}}_3. $$

The ceramic disks with 10-mm diameter and 2-mm thickness were obtained after calcination at 1023 K for 10 h and sintering in air at a temperature of 1273 K for 24 h. The detailed information about the fabrication process has been published elsewhere in [36, 37].

The synthesis of bismuth ferrite BiFeO_3_ with ferroelectric bismuth titanate Bi_4_Ti_3_O_12_ leads to obtaining the Aurivillius Bi_5_Ti_3_FeO_15_ compound. High-purity oxide powders were mixed according to the reaction

4 $$ 5{\mathrm{Bi}}_2{\mathrm{O}}_3+6{\mathrm{Ti}\mathrm{O}}_2+{\mathrm{Fe}}_2{\mathrm{O}}_3\to 2{\mathrm{Bi}}_5{\mathrm{Ti}}_3{\mathrm{Fe}\mathrm{O}}_{15}. $$

The preliminary synthesis of the compound was carried out in air at 1023 K for 10 h while the sintering was performed in air at a temperature of 1253 K for 3 h. The detailed information about the preparation process has been published in our earlier works [38, 39].

The crystalline structure of the sintered samples was investigated by X-ray diffraction (XRD) using the Philips PW3710 diffractometer with CoKα radiation or PANalytical Empyrean diffractometer using CuKα radiation. The phase and structural analysis of the recorded XRD patterns was performed with an X’Pert High Score Plus computer program equipped with the ICDD PDF data base and Rietveld method of the crystalline structure refinement.

**Fig. 1:**
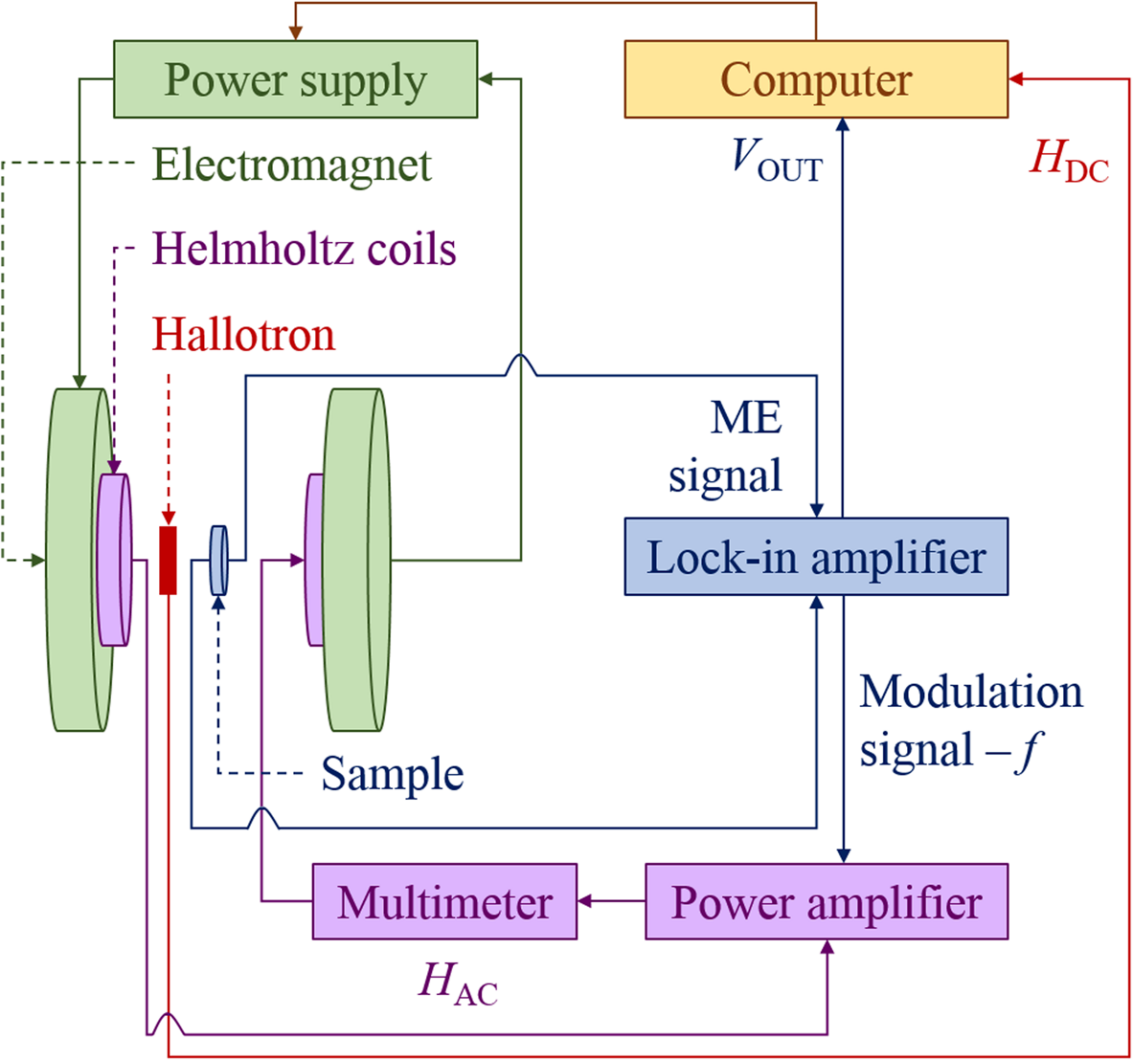
The experimental setup for the dynamic measurement of the magnetoelectric effect [33]

Investigations of the magnetoelectric properties have been performed by the dynamic lock-in technique. The samples were placed in a time-varying DC magnetic field created by an electromagnet and controlled by a programmable DC power source (Fig. 1).

The DC field was modulated by a small sinusoidal AC magnetic field produced by the Helmholtz coils. The voltage induced between the sample surfaces due to the magnetoelectric effect (ME signal) was transmitted to the lock-in amplifier. The amplifier is a key element of the system since it measures and transmits to the computer only this part of the ME signal which is in-phase with the AC modulation field of frequency *f*. Thus, the output signal (*V*_OUT_) measured as a function of the DC field intensity (*H*_DC_) gives an information about the ME effect at a low-AC magnetic field but for different working points of the magnetostrictive samples [33]. The measurements of the *α*_ME_ coefficient were performed by recording *V*_OUT_ under the applied DC magnetic field of the magnitude *H*_DC_ varying within the range of values from 0.1 to 4.5 kOe, while the amplitude *H*_AC_ was constant during the experiment and it was equal to 5 Oe (which is significantly lower than *H*_DC_). The frequency *f* of the AC modulation field was equal to 1 kHz. In the case of (BiFeO_3_)_1 − *x*_–(BaTiO_3_)_*x*_ solid solutions and the Aurivillius Bi_5_Ti_3_FeO_15_ compound, each sample was measured two times, before and after additional electrical poling, in order to verify how the initial polarization of the material affects its magnetoelectric properties. Poling was a process, in which a strong electric field (~3 kV mm^−1^) was applied across the sample at the increased temperature through 1 h before the measurement leading to align the randomly oriented dipoles into one direction. As proved in our earlier work [35], the parallel orientation of the applied electric field versus the magnetic one is better to maximize the value of the *α*_ME_ coefficient. In the case of Bi_1 − *x*_Nd_*x*_FeO_3_ solid solutions, the measurements of *α*_ME_ as a function of *H*_DC_ were performed without initial electrical poling. Moreover, for all the samples, the frequency characteristics were collected in the range of 100 Hz–10 kHz. The measurable magnetoelectric effect was registered above 100 Hz. In the range of 100 Hz–1 kHz, the ME coupling increased rapidly and then stabilized above 1 kHz. Due to the inductance of the Helmholtz coils, the frequency range was limited up to 10 kHz. The kHz range of frequency is justified in the context of possible applications, mainly in energy harvesting devices.

## Results and Discussion

### Results of X-ray Diffraction Studies

The results of X-ray diffraction studies for Bi_1 − *x*_Nd_*x*_FeO_3_ solid solutions and the Aurivillius Bi_5_Ti_3_FeO_15_ compound have been published in our previous works [37–39]. It was found that with an increase of Nd content in Bi_1 − *x*_Nd_*x*_FeO_3_ solid solutions, within the range of *x* = 0.2–0.3, a structural phase transition from rhombohedral to orthorhombic system occurs. The obtained solid solutions were pure and homogeneous samples with very low concentration of secondary phases or without any impurities [37]. In the case of the Bi_5_Ti_3_FeO_15_ compound, a pure Aurivillius phase with orthorhombic lattice (*Fmm2* no. 42 space group) was obtained [38, 39].

**Fig. 2:**
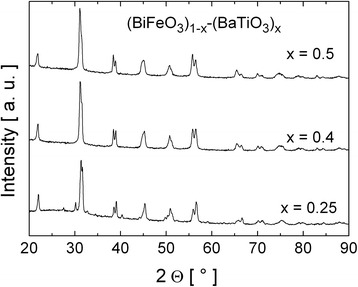
XRD patterns for (BiFeO_3_)_1 − *x*_–(BaTiO_3_)_*x*_ solid solutions with barium titanate concentration *x* = 0.25, 0.4, and 0.5. Low intensity peaks observed for *x* = 0.25 are from the sample holder

Figure 2 presents the results of XRD measurements performed in the current work for (BiFeO_3_)_1 − *x*_–(BaTiO_3_)_*x*_ solid solutions.

The results of the Rietveld refinement method allowed us to conclude that all the obtained samples are no single-phase materials, namely, for *x* = 0.25, the diffractograms revealed two phases, i.e., rhombohedral *R3c* (75 %) and tetragonal *P4mm* (25 %) one. Unfortunately, due to the intensive signal from the sample holder, this estimation of the relative contribution of the phases is uncertain. In the case of *x* = 0.4, three phases were recognized, namely, rhombohedral *R3c* (51 %), cubic *Pm3m* (42 %), and tetragonal *P4mm* (7 %). Finally, XRD pattern for the sample with *x* = 0.5 revealed two phases, i.e., rhombohedral (51 %) and tetragonal (49 %). The obtained result is not fully consistent with the literature data. As reported in [40, 41], the crystallographic symmetry of (BiFeO_3_)_1 − *x*_–(BaTiO_3_)_*x*_ solid solutions changes from rhombohedral for 0 < *x* < 0.3 to cubic for 0.3 < *x* < 0.93 and, finally, to tetragonal for *x* > 0.93. The structural transformation in sintered (BiFeO_3_)_1 − *x*_–(BaTiO_3_)_*x*_ solid solutions from rhombohedral to cubic has been observed by us for the BaTiO_3_ concentration *x* = 0.3 [42]. However, in the case of the analogous solid solutions prepared by us using the mechanical activation method and subsequent isothermal annealing [43], the structural transformation from rhombohedral to cubic symmetry occurred within the region of *x* = 0.4–0.7. Similarly, such gradual transformation was observed for (BiFeO_3_)_1 − *x*_–(SrTiO_3_)_*x*_ solid solutions in Ref. [44], where the structural transformation proceeded for *x* = 0.5–0.7 with the multiphase region in between.

### Magnetoelectric Effect in (BiFeO_3_)_1 − *x*_–(BaTiO_3_)_*x*_ Solid Solutions

**Fig. 3:**
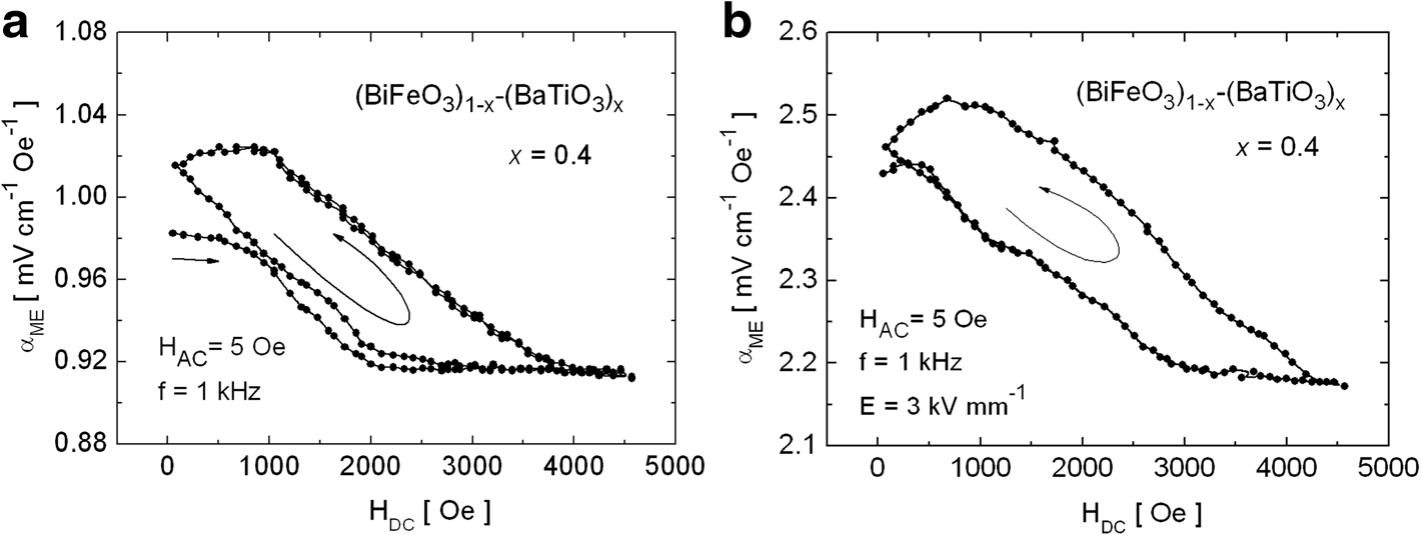
Variation of *α*_ME_ with *H*_DC_ for the (BiFeO_3_)_1 − *x*_–(BaTiO_3_)_*x*_ samples with *x* = 0.4: **a** before electrical poling and **b** after electrical poling at 368 K

The measurements of *α*_ME_ as a function of *H*_DC_ were performed for the set of (BiFeO_3_)_1 − *x*_–(BaTiO_3_)_*x*_ samples with different *x* values. Figure 3 presents the results for *x* = 0.4 as an example.

**Fig. 4:**
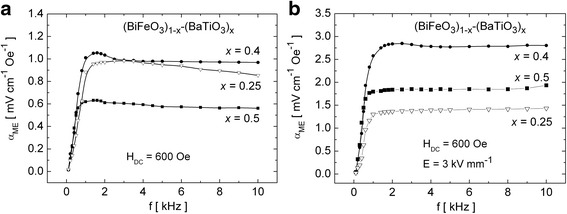
The frequency dependence of the *α*_ME_ coefficient for the (BiFeO_3_)_1 − *x*_–(BaTiO_3_)_*x*_ samples with *x* = 0.25, 0.4, and 0.5: **a** before electrical poling and **b** after electrical poling at 368 K

For all the investigated samples, the curves *α*_ME_(*H*_DC_) do not retrace the same path on the reversal magnetic field when the AC field parameters were fixed. The hysteresis is repeatable (as seen in Fig. 3) and may be connected with the change of orientation of magnetic domains. These results are in consistence with our previous works [35, 42]. Also, the fact that the values of the *α*_ME_ coefficient are generally higher for the samples which were initially polarized electrically has been confirmed. Figure 4 shows the *α*_ME_ dependency on the modulation frequency assuming a constant value of *H*_DC_ = 600 Oe.

The experiment showed that the frequency dependence of the *α*_ME_ coefficient exhibits a strong variability for *f* < 2 kHz. This effect can be caused by the discharge of electric charges accumulated on the surfaces of the investigated samples due to the resistance of the materials [33]. Above the threshold of *f* = 2 kHz, the characteristics are almost flat for all the samples. The flat characteristic is preferred for the practical applications because it allows for a stable operation of the magnetoelectric device in a wide range of frequency. Thus, *f* = 2 kHz would be the optimum frequency for application of the investigated (BiFeO_3_)_1 − *x*_–(BaTiO_3_)_*x*_ solid solutions, for example, as the AC magnetic field sensors.

**Fig. 5:**
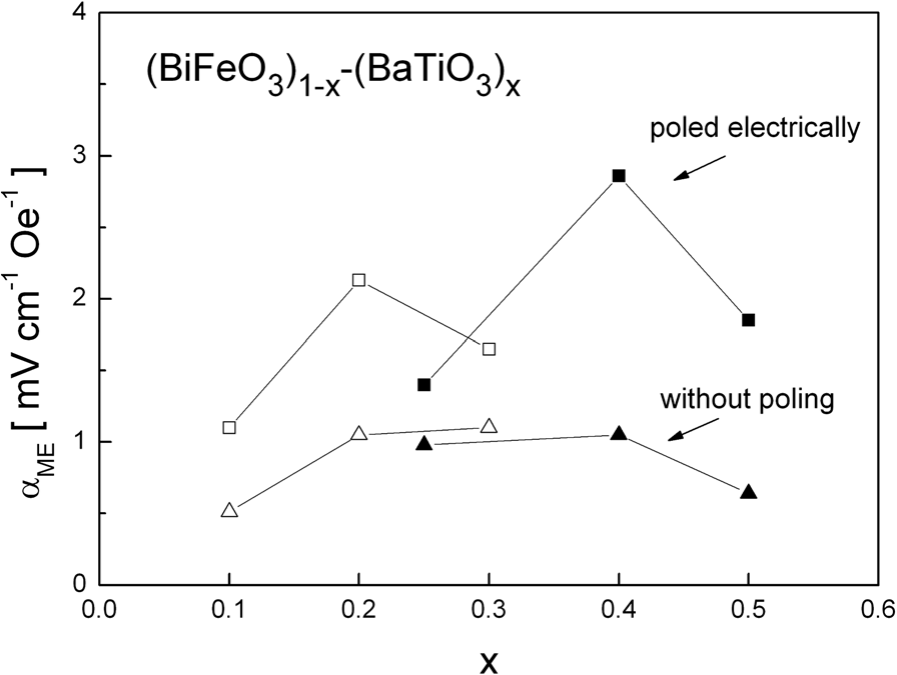
The composition dependence of the *α*_ME_ coefficient for the (BiFeO_3_)_1 − *x*_–(BaTiO_3_)_*x*_ samples; *open symbols*—data from our works [35, 48], *filled symbols*—results in this work

The dependence of the *α*_ME_ coefficient on the concentration of barium titanate is presented in Fig. 5 (the maximum values from the frequency dependencies of *α*_ME_).

The maximum value of *α*_ME_ = 2.84 mVcm^−1^ Oe^−1^ achieved in this study for the electrically poled sample with *x* = 0.4 is higher even up to 85 % when compared with the previous results for the sample with *x* = 0.3 (*α*_ME_ = 1.53 mVcm^−1^ Oe^−1^) [35] and about three times higher than the results reported by Yang for 0.75BiFeO_3_–0.25BaTiO_3_ (*α*_ME_ = 0.87 mVcm^−1^ Oe^−1^) [34]. It may be seen that the value of the *α*_ME_ coefficient first increases with an increase of *x* which is connected with suppression of the cycloidal spin structure in BiFeO_3_. For *x* = 0.2–0.4, the magnitude of magnetoelectric coupling is practically constant and then decreases. Electrical poling allows increasing the value of *α*_ME_ by 2–3 times what may be caused with the presence of the tetragonal phase. It may be supposed that multiphase structure of (BiFeO_3_)_1 − *x*_–(BaTiO_3_)_*x*_ solid solutions for *x* = 0.25–0.5 promotes greater magnetoelectric coupling, especially after initial electrical poling of the samples.

### Magnetoelectric Effect in Bi_1 − *x*_Nd_*x*_FeO_3_ Solid Solutions

**Fig. 6:**
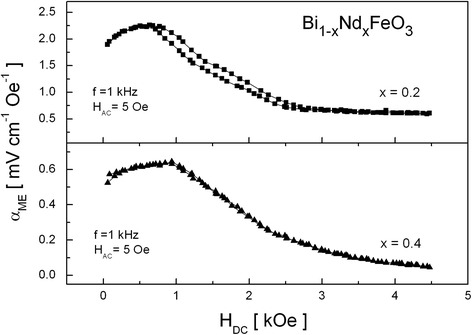
Variation of *α*_ME_ with *H*_DC_ for Bi_1 − *x*_Nd_*x*_FeO_3_ solid solutions with *x* = 0.2 and *x* = 0.4

In the case of Bi_1 − *x*_Nd_*x*_FeO_3_ solid solutions, *α*_ME_ reaches the maximum value for 1 kOe and then quickly drops. Figure 6 presents the curves *α*_ME_(*H*_DC_) for *x* = 0.2 and 0.4 as an example. It was observed that weak hysteresis in *α*_ME_(*H*_DC_) dependence occurred in the range of neodymium concentration *x* = 0.1–0.3, while for *x* ≥ 0.4, the hysteresis disappeared.

**Fig. 7:**
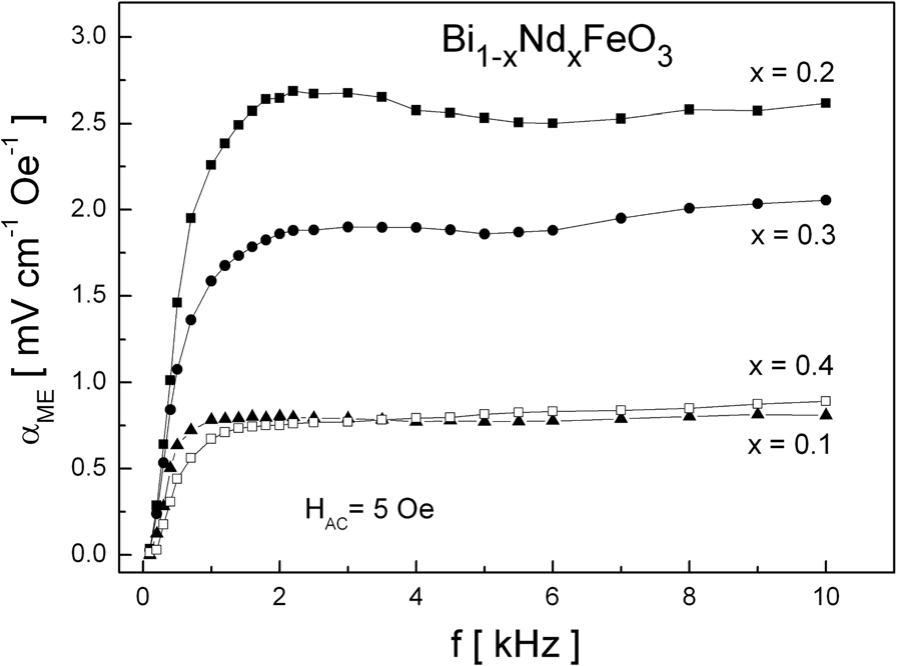
The frequency dependence of the *α*_ME_ coefficient for Bi_1 − *x*_Nd_*x*_FeO_3_ solid solutions with *x* = 0.1–0.4

The frequency dependence of the *α*_ME_ coefficient for Bi_1 − *x*_Nd_*x*_FeO_3_ solid solutions is presented in Fig. 7 for various *x*. The dependence shows similar behavior as for (BiFeO_3_)_1 − *x*_–(BaTiO_3_)_*x*_ solid solutions, the *α*_ME_ coefficient reaches maximum for 1–2 kHz and then saturates.

It may be seen that *α*_ME_ initially increases with an increase of *x* and reaches the maximum value *α*_ME_ ~ 2.7 mVcm^−1^ Oe^−1^ for *x* = 0.2. This increase is attributed to quenching of the cycloidal spin structure of BiFeO_3_. Further increase of *x* causes monotonical decrease of *α*_ME_, and for *x* = 0.7–0.9, the ME signal was unmeasurably small. The changes of magnetoelectric coupling coefficient in Bi_1 − *x*_Nd_*x*_FeO_3_ solid solutions are accompanied with structural transformation from rhombohedral to orthorhombic system for *x* = 0.2–0.3.

### Magnetoelectric Effect in the Aurivillius Bi_5_Ti_3_FeO_15_ Compound

**Fig. 8:**
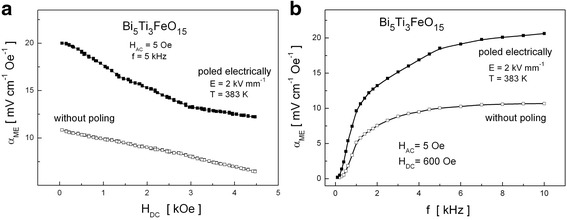
**a** Variation of *α*_ME_ with *H*_DC_ and **b** frequency dependence of the *α*_ME_ coefficient for the Bi_5_Ti_3_FeO_15_ compound without and with electrical poling

As proved by XRD, Mössbauer spectroscopy, and magnetic measurements, the Bi_5_Ti_3_FeO_15_ Aurivillius compound is a single-phase paramagnetic material within the broad temperature range 2–350 K (our earlier articles [38, 39, 45]). Despite being paramagnetic at room temperature, i.e., in the absence of magnetic long-range order, the sample shows magnetoelectric coupling (Fig. 8). Measurements show that the *α*_ME_ coefficient decreases almost linearly with an increase of *H*_DC_ and no hysteretic behavior was observed (Fig. 8a). The dependence of the *α*_ME_ on *f* shows an increase of the magnitude of ME response of the sample within relatively broad range of frequency 0–5 kHz (Fig. 8b). Above the threshold of *f* = 5 kHz, the characteristics are almost flat. As seen in Fig. 8, electrical poling allowed us to double the value of *α*_ME_.

As shown in the latest theoretical first-principles calculations, for the four-layer Aurivillius-phase Bi_5_Ti_3_FeO_15_ [46], there is a strong antiferromagnetic coupling between Fe^3+^ cations in the nearest-neighbor positions, characteristic of the superexchange interaction between *d*^5^ cations (Fe-O-Fe). Moreover, the coupling between further neighbors is rather weak and becomes negligible beyond second neighbors. Due to the relatively low concentration of magnetic cations in Bi_5_FeTi_3_O_15_ and the short range of the magnetic superexchange interaction, it is unclear whether magnetic long-range order can occur in this system. Unexpectedly, our research shows that the magnetoelectric coupling in the Bi_5_Ti_3_FeO_15_ compound is relatively high. We suppose that the ME coupling in this single-phase material may be connected with short-range magnetic ordering of Fe-rich nano-regions, which mimic BiFeO_3_. Similar interpretation was reported for Bi_5_Ti_3_FeO_15_ thin films grown by pulsed layer deposition with a robust magnetoelectric coupling of 400 mVcm^−1^ Oe^−1^ [47].

## Conclusions

On the basis of the performed studies, it may be stated that the best candidate for the single-phase room-temperature multiferroic material is the Aurivillius Bi_5_FeTi_3_O_15_ compound. Using solid-state sintering method, a pure single phase was successfully obtained. The ME effect in the Bi_5_FeTi_3_O_15_ compound is present despite the absence of long-range magnetic order, and relatively strong ME signal cannot be caused by tiny inclusions of other phases. The existence of the magnetoelectric coupling in the paramagnetic Aurivillius Bi_5_FeTi_3_O_15_ compound and explanation of mechanism of this coupling is open for future studies.

In the case of (BiFeO_3_)_1 − *x*_–(BaTiO_3_)_*x*_ and Bi_1 − *x*_Nd_*x*_FeO_3_ solid solutions, the ME effect is connected with suppression of the cycloidal spin structure of BiFeO_3_. The magnitude of magnetoelectric coupling depends on the concentration of barium titanate and neodymium in the bismuth ferrite structure. The maximum values of the magnetoelectric coupling coefficient were obtained for the compositions within the region of structural transformations.

As it was shown, an initial electrical poling allows increasing the ME effect 2–3 times. The frequency dependencies of *α*_ME_ have similar shapes for all the investigated materials and allowed us to determine the optimum frequency range, i.e., *f* ≥ 2 kHz for (BiFeO_3_)_1 − *x*_–(BaTiO_3_)_*x*_ and Bi_1 − *x*_Nd_*x*_FeO_3_ solid solutions and *f* ≥ 5 kHz for the Aurivillius Bi_5_FeTi_3_O_15_ compound. Moreover, no hysteretic behavior was observed in *α*_ME_(*H*_DC_) dependence in the case of single-phase materials.
